# Baseline patient-reported symptom networks and early risk stratification for six-month symptom burden in newly diagnosed glioma: a prospective cohort study

**DOI:** 10.3389/fpubh.2026.1830043

**Published:** 2026-06-12

**Authors:** Genxiao Ding, Xiaojing Meng, Ran Duan, Dong Song, Chuanxi Wang, Fengping Zhang, Jie Mei

**Affiliations:** 1 Department of Neurosurgery, Henan Provincial People’s Hospital, Zhengzhou, China; 2 Neurosurgical Critical Care Unit, Henan Provincial People’s Hospital, Zhengzhou, China

**Keywords:** glioma, patient-reported outcomes, prospective cohort, risk stratification, supportive care, symptom burden, symptom network

## Abstract

**Objective:**

To characterize the baseline patient-reported symptom network in newly diagnosed glioma and to develop a baseline-only model for predicting high symptom burden at 6 months, with the aim of informing earlier symptom surveillance and risk-stratified supportive care.

**Methods:**

In a prospective cohort of 142 adults with histologically confirmed glioma, 15 patient-reported symptoms were rated at baseline using 0–10 numeric rating scales. Baseline symptom networks were estimated using Gaussian graphical modeling with graphical LASSO regularization and EBIC model selection. Edge accuracy and centrality stability were evaluated using nonparametric bootstrap and case-dropping procedures. High symptom burden at 6 months was defined using an upper-quartile rule based on the cohort distribution of the 6-month total symptom score. A random forest model incorporating baseline symptoms, clinical variables, and psychosocial factors was trained using a stratified split and internally validated in an independent test set.

**Results:**

The baseline network was moderately dense (38 edges; density = 0.36) and showed prominent cross-domain interdependencies. Speech difficulty and headache had the highest strength centrality, whereas betweenness and closeness showed lower stability. The random forest model achieved an AUC of 0.947 (95% CI, 0.888–1.000), and the leading predictors were baseline anxiety, depression, memory impairment, pain, fatigue, and age.

**Conclusion:**

Symptom burden in newly diagnosed glioma appears to arise from an interconnected patient-reported symptom pattern in which emotional, cognitive, physical, and neurological symptoms interact closely. Baseline multidimensional assessment and early risk stratification may help identify patients who warrant closer symptom surveillance and supportive care during the initial treatment course. These findings provide a basis for further external validation.

## Introduction

Adult gliomas remain among the most challenging solid malignancies because they combine aggressive biology with direct injury to functional brain networks (1). Although survival remains a major focus, quality of life has emerged as an equally important endpoint, particularly given the persistence of symptoms across the disease course. Patients frequently report psychological distress, fatigue, sleep problems, pain, and cognitive impairment. These symptoms impair treatment tolerance, undermine rehabilitation, and increase caregiver burden (2–4). Conventional symptom assessment, whether during outpatient follow-up or neurosurgical hospitalization, often treats symptoms as separate entities. However, symptom science increasingly supports a systems perspective: symptoms tend to cluster, and changes in one complaint can trigger changes in others. This interdependence is clinically relevant in glioma, where neuroanatomical disruption, neuroinflammation, corticosteroid exposure, antiseizure medications, and radiochemotherapy interact in ways (5–8) that are difficult to disentangle using single-symptom approaches.

Network analysis provides a framework in which symptoms are represented as nodes connected by edges that quantify conditional associations (9). In this framework, centrality reflects the relative prominence of symptoms within the network (10, 11). In oncology, network approaches have highlighted the centrality of fatigue and mood symptoms, but glioma-specific evidence remains limited and may differ because of the neurological substrate of the disease.

Alongside descriptive network approaches, there is also a practical need for prediction. Clinicians and nurses need tools that can identify patients who are likely to develop severe symptom burden in the months following diagnosis, when adjuvant therapy begins and supportive care needs intensify. From a public health and care-delivery perspective, early identification of patients at elevated risk may help target symptom surveillance, supportive interventions, and follow-up resources more efficiently. Machine-learning methods can integrate heterogeneous predictors and capture nonlinear interactions, thereby complementing traditional regression approaches (12, 13). However, most machine-learning applications in glioma have focused on survival, radiomics, or molecular classification, leaving symptom risk stratification and patient-reported outcome prediction underdeveloped (14–16).

We conducted a prospective cohort study of 142 newly diagnosed glioma patients to address two aims. First, we characterized the baseline symptom network to identify central symptoms and robust intersymptom associations that may guide more integrated symptom management. Second, we developed and internally validated a baseline-only prediction model for high symptom burden at 6 months to support early risk stratification, patient-reported outcome surveillance, and supportive care planning at the time when treatment pathways are established.

## Materials and methods

### Study design and participants

This prospective observational cohort enrolled consecutive adults with newly diagnosed, histologically confirmed glioma at Henan Provincial People’s Hospital since February 2022. Baseline assessments were completed at diagnosis, and outcome ascertainment was performed at the scheduled 6-month follow-up. The study was designed to link baseline symptom-system structure with near-term symptom burden during the early treatment course, with an emphasis on patient-reported symptom monitoring and supportive care planning.

### Assessment schedule and procedures

Assessments were performed at two standardized time points: baseline (within 2 weeks of diagnosis, before or immediately after surgery) and 6 months. Trained neurosurgical nurses administered the symptom ratings and psychosocial questionnaires in person whenever possible; for patients who were unable to return, the 6-month assessment was completed by structured telephone interview using the same instruments.

### Symptom measures

We assessed 15 symptoms commonly reported by glioma patients—nausea, sleep disturbance, weakness, speech difficulty, drowsiness, memory impairment, attention deficit, headache, seizure, appetite loss, fatigue, anxiety, depression, pain, and vision problems—using 0–10 numeric rating scales (0 = none; 10 = worst imaginable). Symptoms were grouped *a priori* into emotional (anxiety, depression), cognitive (memory impairment, attention deficit), physical (fatigue, weakness, appetite loss, nausea), and neurological (headache, seizure, pain, drowsiness, sleep disturbance, speech difficulty, and vision problems) domains for descriptive purposes. This pragmatic patient-reported symptom inventory was designed for routine neurosurgical follow-up and symptom surveillance, and it is less granular than dedicated brain tumor-specific instruments such as the MDASI-BT (17, 18).

### Clinical and psychosocial variables

Baseline clinical variables included age, sex, tumor grade, tumor size (recorded as a three-level ordinal variable derived from preoperative MRI), tumor location (frontal, temporal, parietal, occipital, or multifocal), and Karnofsky Performance Status (KPS) score (19). Psychosocial measures included resilience (Chinese version of the Connor-Davidson Resilience Scale; 0–100) and perceived social support (Multidimensional Scale of Perceived Social Support adapted for Chinese populations; 1–7) (20, 21). Treatment exposures (extent of resection, radiotherapy, chemotherapy) were recorded for cohort description but were not included in the baseline prediction model, so that the model reflected information available at diagnosis.

### Outcome definition

The primary outcome was high symptom burden at 6 months. For each participant, total symptom burden at 6 months was calculated as the sum of the 15 symptom-severity ratings recorded at the 6-month assessment, with each item scored from 0 to 10 and the total score ranging from 0 to 150. Patients whose total score fell in the highest segment of the cohort distribution, defined using an upper-quartile rule, were classified as having high symptom burden. Because the summed score is discrete, ties around the quartile boundary were possible, and the final proportion classified as having high burden could therefore exceed 25%. The 6-month time point was chosen because it reflects a stage at which the effects of postoperative recovery and adjuvant treatment are both evident in patients’ symptom experience.

### Statistical analysis

#### Symptom network estimation

Baseline symptom networks were estimated using Gaussian graphical models, in which edges represent partial correlations between symptoms after conditioning on all other symptoms. To reduce false-positive edges, we applied graphical LASSO regularization with Extended Bayesian Information Criterion (EBIC) model selection (EBIC tuning parameter set at the recommended default for psychometric networks). Networks were visualized with qgraph (version 1.9.8). Centrality indices were calculated for strength, betweenness, and closeness. To assess robustness, we used nonparametric bootstrapping (1,000 iterations) to estimate confidence intervals for edge weights and correlation-stability (CS) coefficients for centrality indices; CS > 0.2 was considered indicative of interpretable stability (22–24). We prespecified the regularization and stability-evaluation steps to limit analytic flexibility and to keep interpretation focused on associations that were reproducible under resampling.

#### Predictive modeling and validation

Participants were randomly divided into a training set (70%, *n* = 99) and an independent testing set (30%, *n* = 43), with stratification by outcome status. Missing data were limited and were handled using random-forest imputation. Candidate predictors included baseline symptom severity, clinical variables, and psychosocial measures. A random forest classifier with 500 trees was fitted, and the number of candidate variables sampled at each split was tuned by 10-fold cross-validation within the training set (25). Model performance was evaluated in the testing set using the receiver operating characteristic curve and the area under the curve, with 95% confidence intervals estimated by nonparametric bootstrap resampling (26). Because the model was intended as an early-warning and risk-stratification tool, we focused primarily on discrimination; calibration, clinical utility, and threshold-based performance measures should be examined in external validation studies (27, 28).

#### Follow-up and missing-data handling

Six-month assessments were completed during clinic visits or by structured telephone interviews conducted by trained neurosurgical nurses using the same symptom instruments as at baseline. Analyses were based on participants with available 6-month outcome data. For predictive modeling, we performed a complete-case analysis for the primary endpoint and used stratified sampling to preserve the event proportion in the training and testing sets. Uncertainty in model discrimination was quantified using nonparametric bootstrap resampling to derive confidence intervals for the AUC.

## Results

### Participant characteristics

The cohort comprised 142 patients (mean age 55.0 years, SD 7.5; 70 male, 49.3%). Tumor grades were distributed as follows: grade I, 39 (27.5%); grade II, 32 (22.5%); grade III, 33 (23.2%); and grade IV, 38 (26.8%). Tumor size was recorded on a three-level ordinal scale (1–3), with 43 (30.3%), 42 (29.6%), and 57 (40.1%) in categories 1, 2, and 3, respectively. Functional status was generally preserved (KPS 84.6 ± 5.5). Mean resilience score was 70.6 ± 7.9, and mean perceived social support score was 1.94 ± 0.84. At 6 months, 50 patients (35.2%) met the criteria for high symptom burden. Baseline characteristics are summarized in Table 1.

**Table 1:** Baseline characteristics of the study cohort (*N* = 142).

Characteristic	Value
Demographics	
Age, years	55.0 ± 7.5
Male sex	70/142 (49.3%)
Tumor characteristics	
Tumor grade I	39 (27.5%)
Tumor grade II	32 (22.5%)
Tumor grade III	33 (23.2%)
Tumor grade IV	38 (26.8%)
Tumor size category 1 (ordinal 1 to 3)	43 (30.3%)
Tumor size category 2 (ordinal 1 to 3)	42 (29.6%)
Tumor size category 3 (ordinal 1 to 3)	57 (40.1%)
Functional and psychosocial measures	
Karnofsky Performance Status (KPS)	84.6 ± 5.5
Resilience score	70.6 ± 7.9
Perceived social support score	1.94 ± 0.84
Outcome	
High symptom burden at 6 months	50/142 (35.2%)
Baseline symptom severity (0 to 10)	
Fatigue	4.97 ± 1.77
Pain	5.03 ± 1.82
Sleep	5.12 ± 1.87
Anxiety	5.06 ± 1.87
Depression	4.94 ± 1.80
Memory	5.05 ± 1.80
Attention	5.10 ± 1.82
Nausea	5.08 ± 1.75
Appetite	5.07 ± 1.76
Weakness	5.05 ± 1.98
Headache	5.04 ± 1.80
Seizure	4.87 ± 1.70
Speech	4.93 ± 1.73
Vision	5.01 ± 1.77
Drowsiness	5.10 ± 1.89

Values are mean ± SD or *n* (%). Symptom severity was rated on a 0 to 10 scale. Tumor size was stored as an ordinal variable (1 to 3) in the source dataset. KPS, Karnofsky Performance Status; SD, standard deviation.

### Baseline symptom network structure

**Figure 1:**
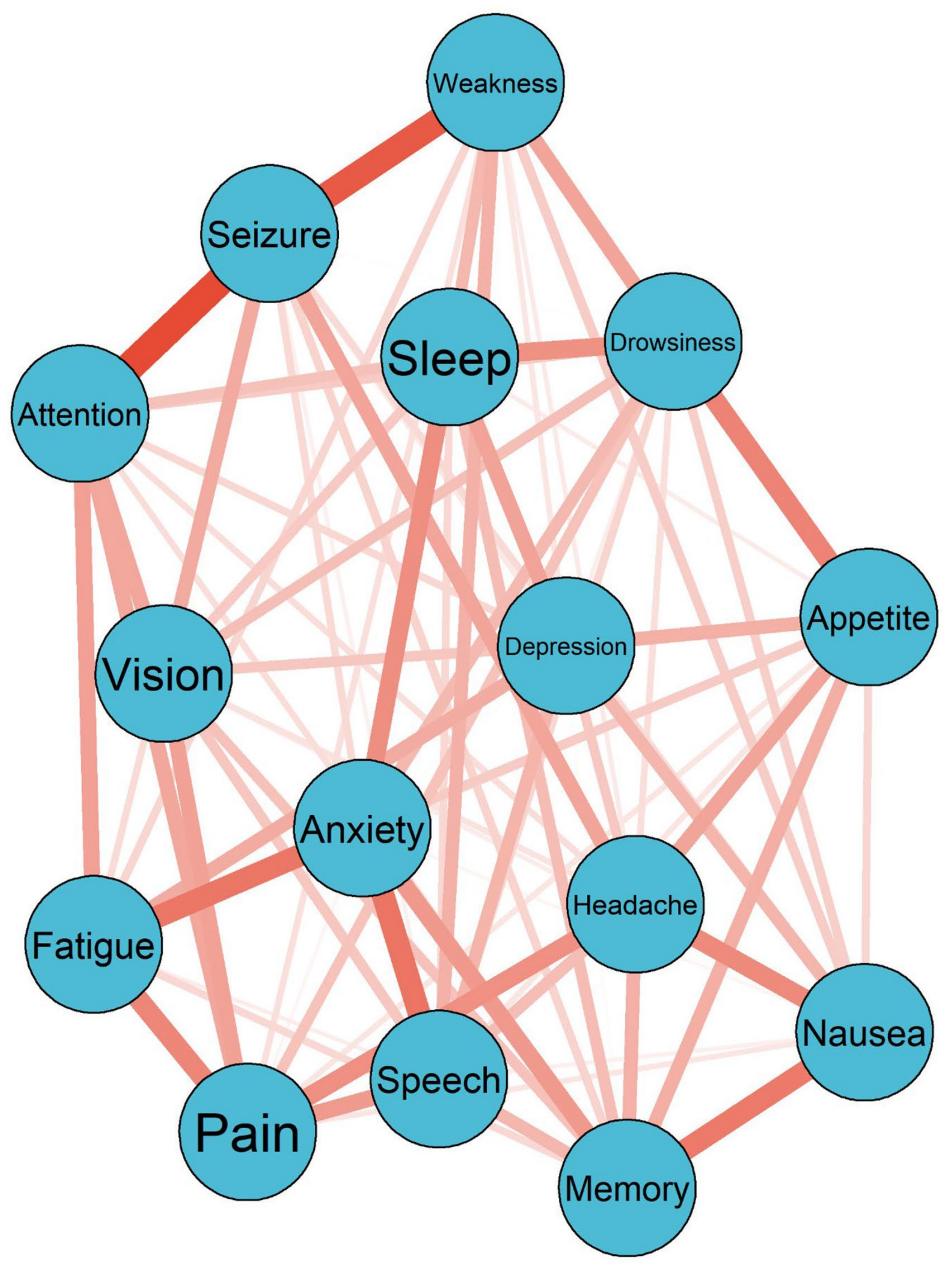
Baseline symptom network in glioma patients (*N* = 142). Network visualization of 15 symptom nodes, with edges representing partial correlations estimated by Gaussian graphical modeling with graphical LASSO regularization. Node size reflects strength centrality; thicker edges indicate stronger associations.

**Figure 2:**
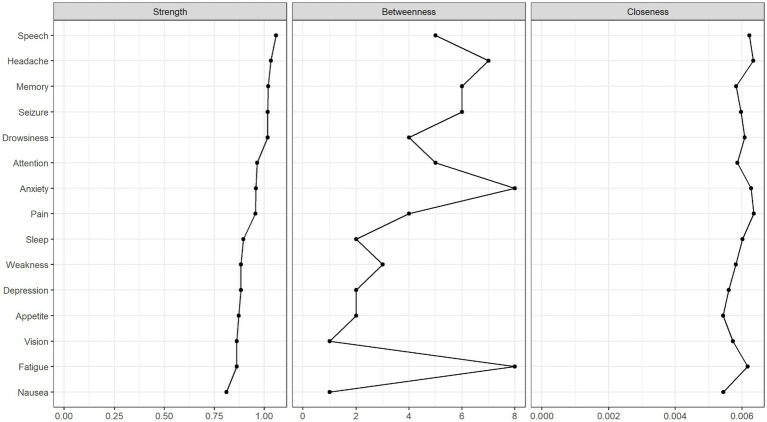
Centrality profiles of the baseline symptom network. Strength, betweenness, and closeness centrality indices are plotted for each symptom node to summarize relative connectedness across the network.

**Figure 3:**
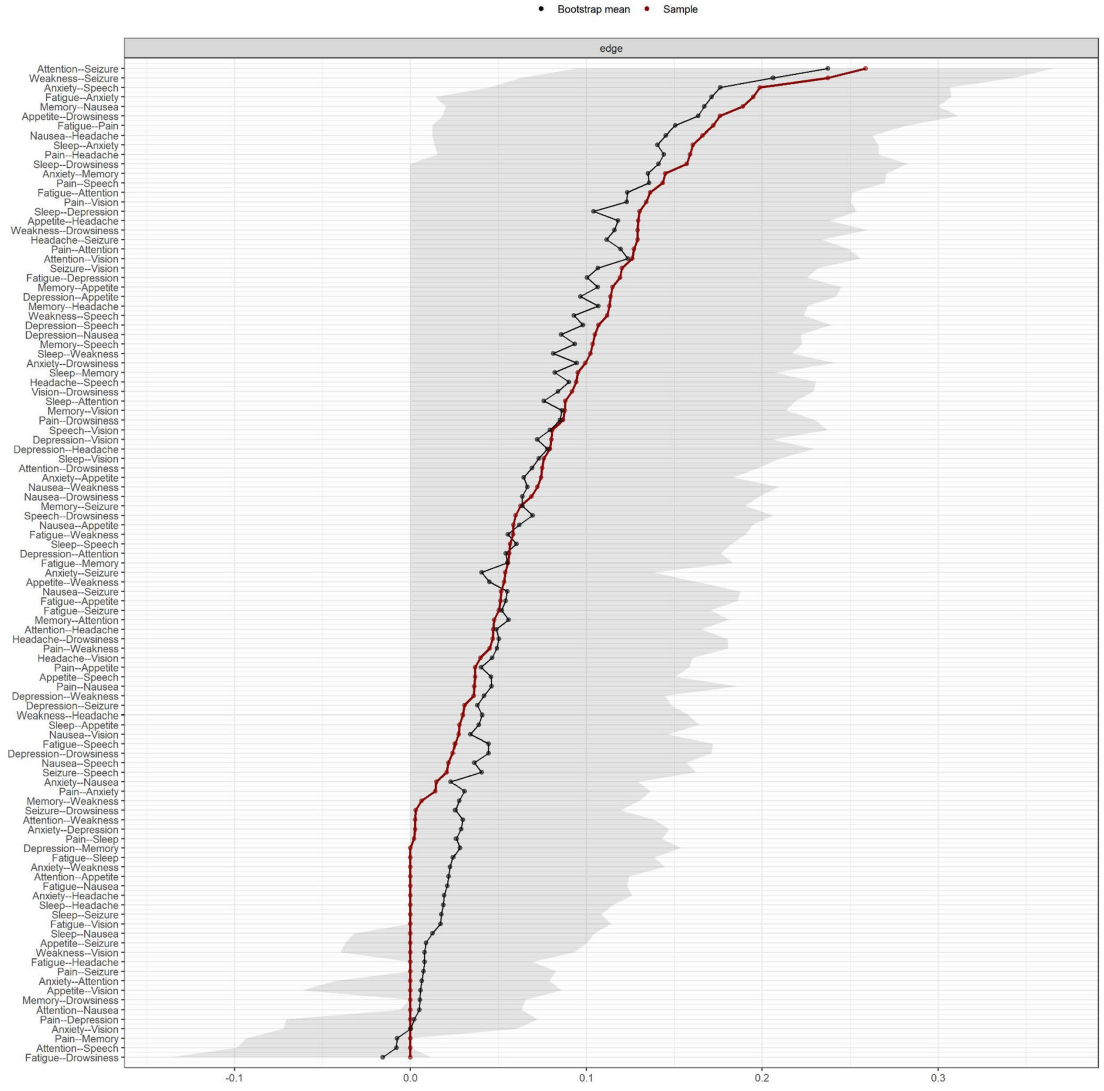
Bootstrap confidence intervals for edge weights. Nonparametric bootstrap results depict the sampling variability of estimated partial correlations, supporting interpretation of robust versus uncertain symptom associations.

**Figure 4:**
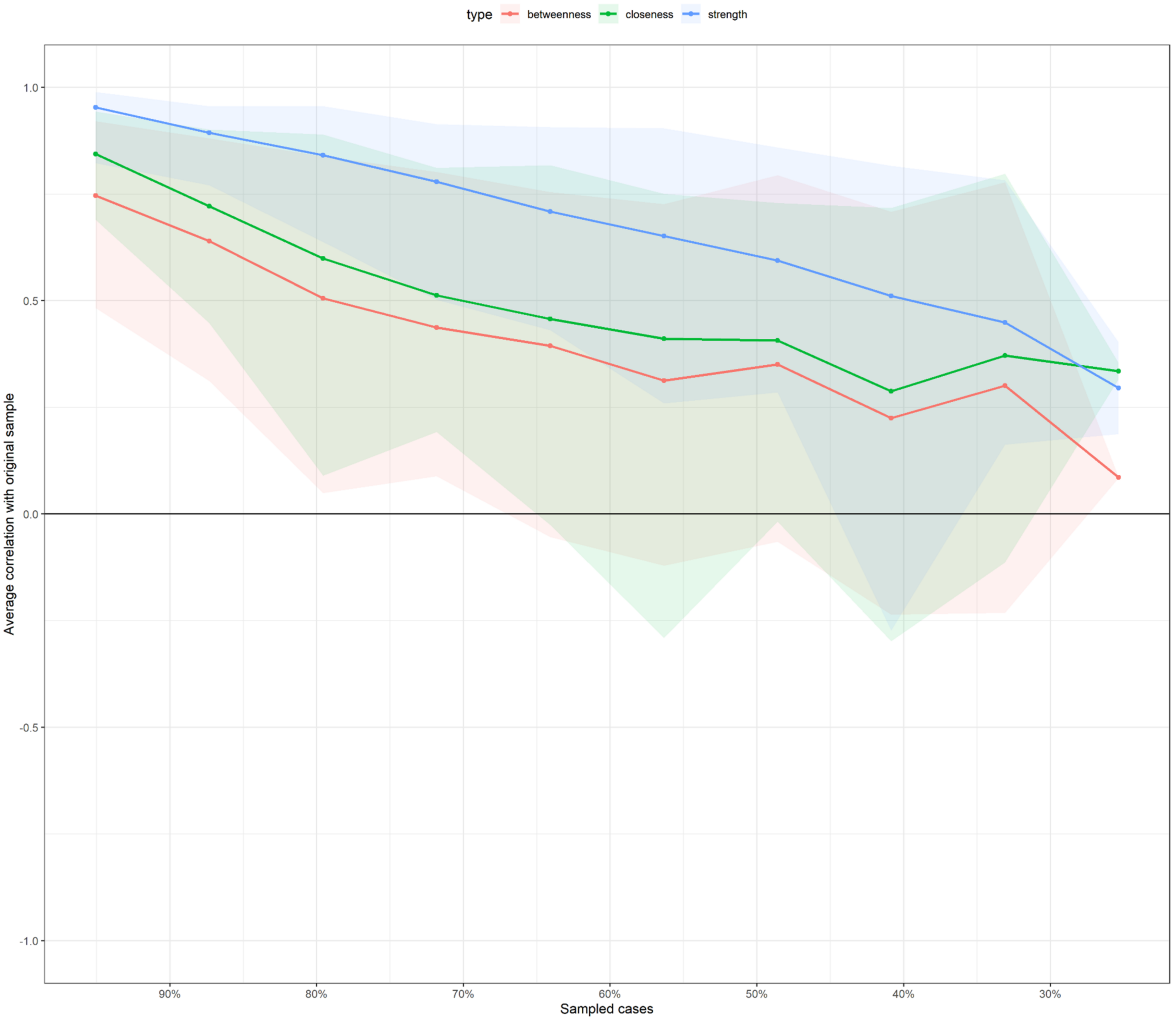
Case-dropping bootstrap for centrality stability. Correlations between centrality indices in subset samples and the original network are shown across increasing proportions of case removal, with shaded uncertainty bands.

The baseline symptom network included 15 nodes connected by 38 non-zero edges (density = 0.36) (Figures 1, 2). Strength centrality was highest for speech difficulty (1.06) and headache (1.03), with memory impairment (1.02), seizure (1.02), and drowsiness (1.02) also ranking prominently. Betweenness was highest for weakness (27), anxiety (26), and depression (21), with these symptoms occupying more cross-network positions in the descriptive centrality profile. To evaluate network robustness, we examined the sampling variability of edge estimates and the stability of centrality indices. The nonparametric bootstrap indicated that many small-magnitude edges were uncertain, whereas a subset of associations showed relatively narrow confidence intervals (Figure 3). Case-dropping analyses suggested modest stability for strength centrality (CS = 0.204) and poor stability for closeness and betweenness (Figure 4). Taken together, the bootstrap results indicate that the network is most informative at the level of robust edges and overall topology.

### Symptom correlation patterns

**Figure 5:**
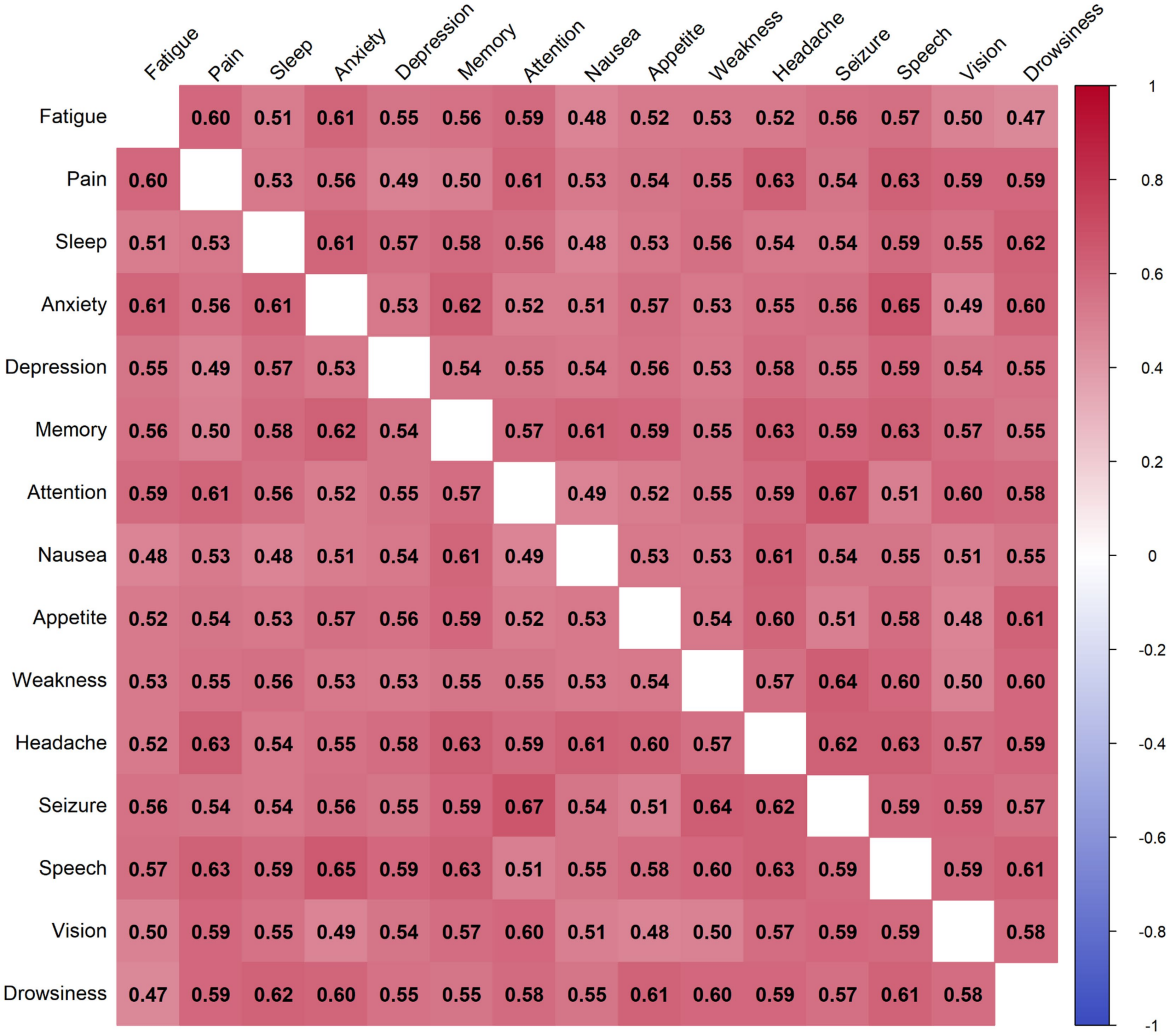
Symptom correlation matrix at baseline.

Zero-order correlations supported the network structure and provided an intuitive overview of symptom co-occurrence (Figure 5). The strongest correlations were observed for attention deficit and seizure (*r* = 0.67), anxiety and speech difficulty (*r* = 0.65), weakness and seizure (*r* = 0.64), and pain and headache (*r* = 0.63). Emotional symptoms were also moderately correlated, with anxiety and depression showing a correlation of 0.53. Cognitive complaints covaried with neurological and arousal-related symptoms, supporting a multidomain symptom pattern at diagnosis (27).

Beyond the strongest correlations, fatigue correlated moderately with weakness (*r* = 0.54) and depression (*r* = 0.57), supporting its multifactorial nature. Memory impairment and attention deficit were both strongly associated with drowsiness (memory impairment and drowsiness, *r* = 0.53; attention deficit and drowsiness, *r* = 0.59), whereas focal symptoms such as vision problems and speech difficulty showed weaker correlations with several other symptoms. Overall, these patterns suggest that global neurobehavioral symptoms and emotional distress may cluster more closely than location-specific focal deficits.

### Community structure

**Figure 6:**
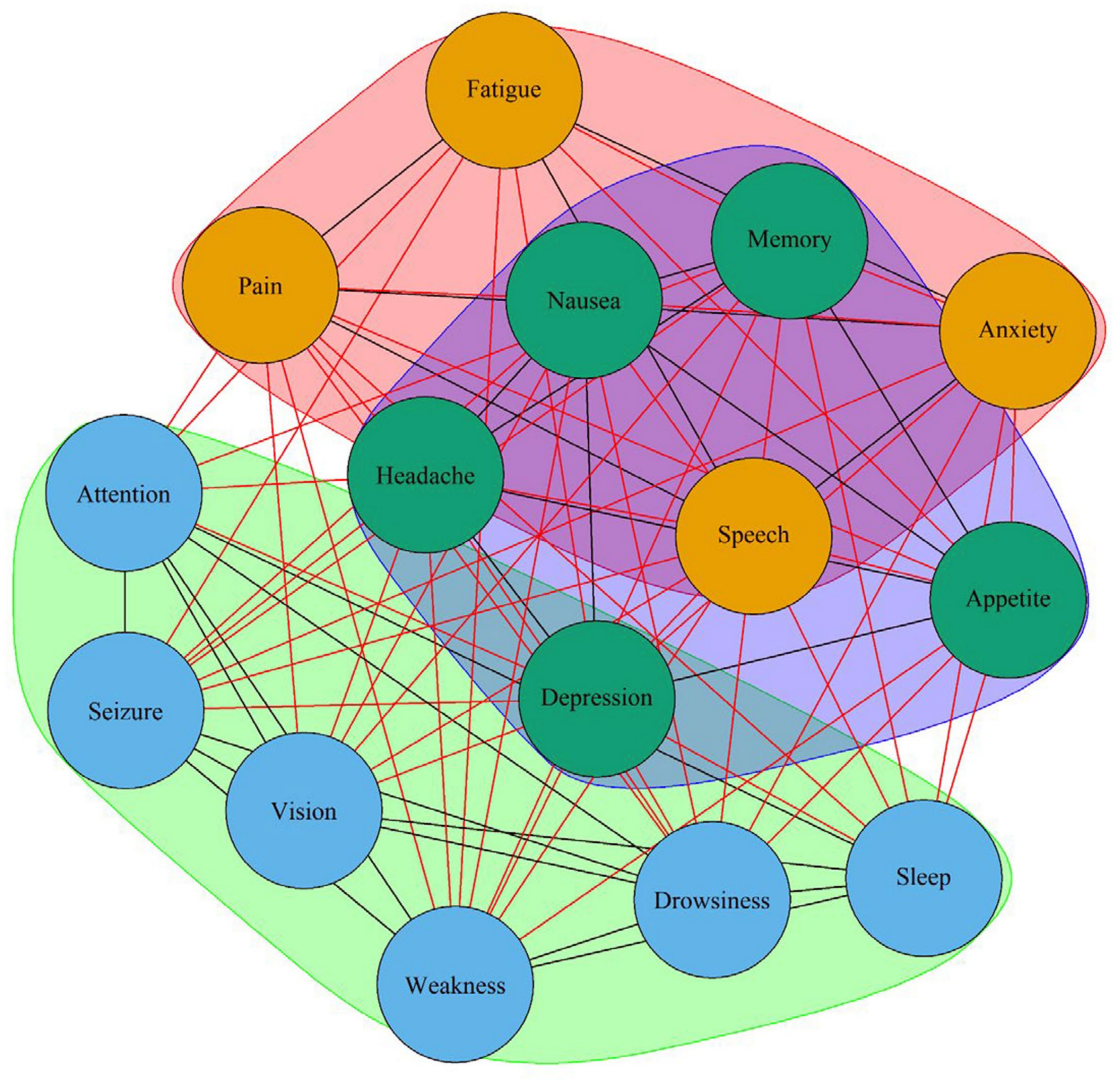
Community structure of the symptom network. Communities identified from the baseline network are visualized with colored groupings and convex hulls, illustrating modular organization and cross-community links.

Community detection indicated a modular yet overlapping organization of the symptom network (Figure 6). A neurological and arousal-related community comprising attention deficit, seizure, vision problems, weakness, drowsiness, and sleep disturbance appeared tightly connected. Another community included fatigue, pain, speech difficulty, and anxiety, while headache, nausea, depression, memory impairment, and appetite loss formed a partially overlapping group. The overlap among communities suggests that symptom domains were not clearly separated. Depression and headache also linked across communities, consistent with their relatively central positions in the network.

### Prediction of high symptom burden at 6 months

**Figure 7:**
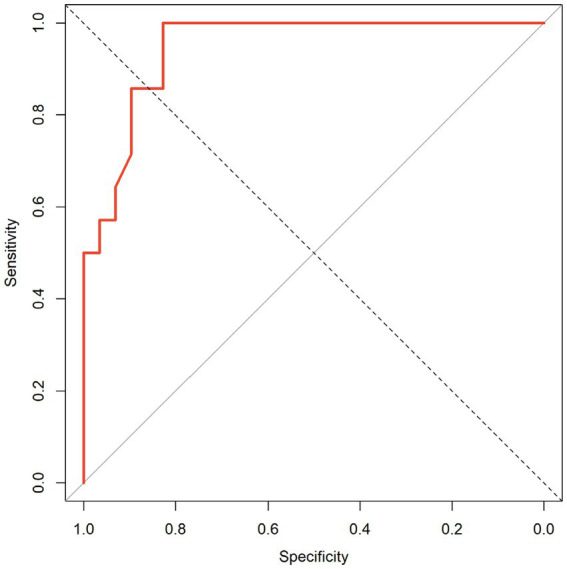
Receiver operating characteristic curve for predicting high symptom burden at 6 months.

**Figure 8:**
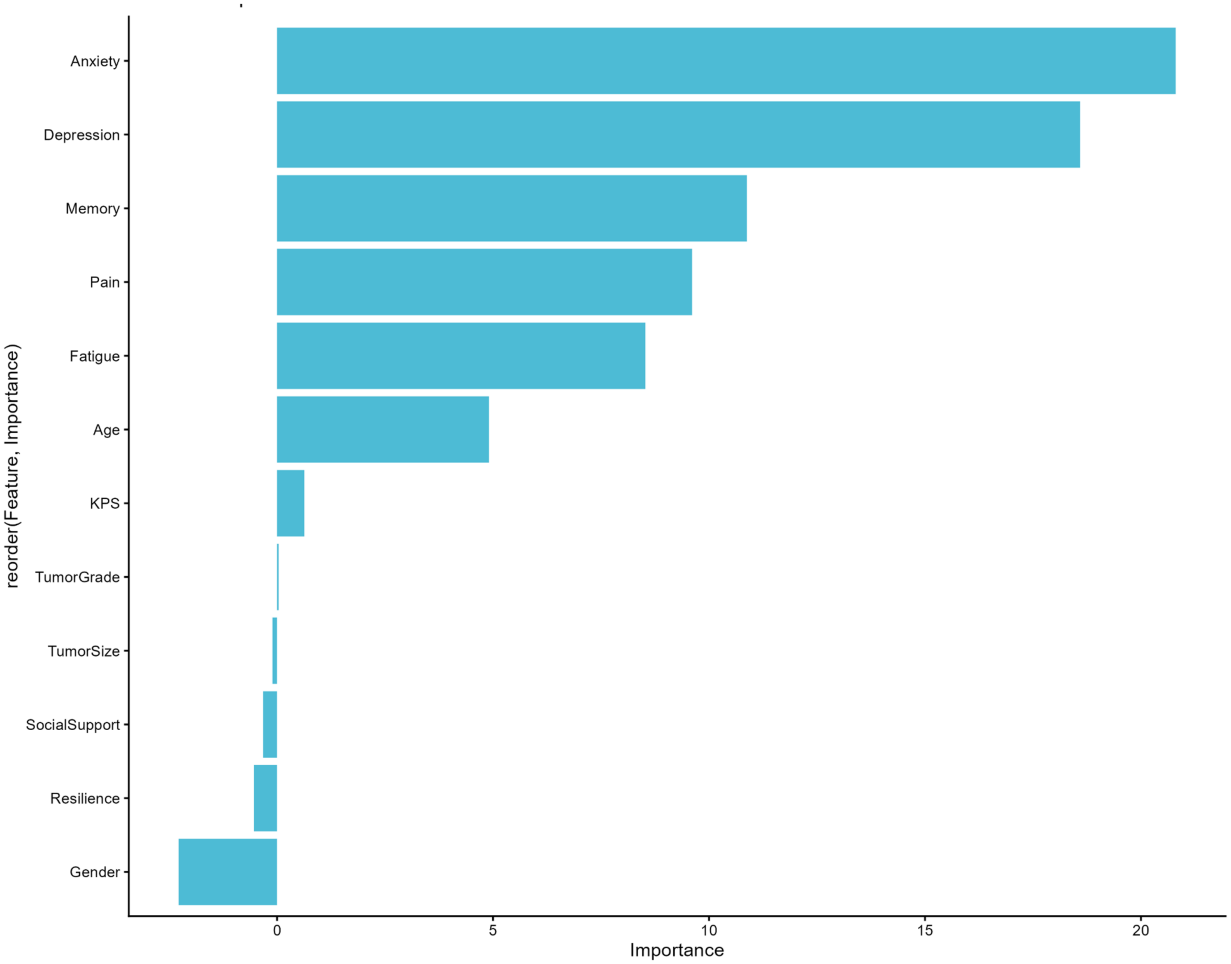
Feature importance for predicting high symptom burden (random forest).

In the training set (*n* = 99), 35 patients (35.4%) met the criteria for high symptom burden at 6 months; the independent testing set included 43 patients, 15 of whom (34.9%) had high symptom burden. The random forest achieved an AUC of 0.947 (95% CI, 0.888–1.000) in the testing set (Figure 7), indicating strong discrimination between patients with high and lower symptom burden in this dataset. Variable importance was driven mainly by baseline symptom severity. Anxiety had the highest importance (20.8), followed by depression (18.59), memory impairment (10.88), pain (9.61), and fatigue (8.53) (Figure 8; Table 2). Age also made a modest contribution (4.91). In contrast, clinical and psychosocial variables contributed little to the model, with only limited importance for KPS (0.63) and tumor grade (0.04), while tumor size (−0.11), social support (−0.32), resilience (−0.53), and sex (−2.28) showed minimal or negligible contribution.

**Table 2:** Random forest predictors of 6-month high symptom burden (*N* = 142).

Predictor	Importance (mean decrease in Gini)
Anxiety	20.80
Depression	18.59
Memory	10.88
Pain	9.61
Fatigue	8.53
Age	4.91
KPS	0.63
Tumor grade	0.04
Tumor size	−0.11
Social support	−0.32
Resilience	−0.53
Gender	−2.28

## Discussion

Symptoms in newly diagnosed glioma did not present as isolated complaints. They formed a moderately dense network spanning affective, cognitive, physical, and neurological domains, and baseline information was sufficient to identify patients at increased risk of substantial symptom burden at 6 months. Symptom assessment at diagnosis therefore needs to look beyond individual complaints and consider the broader pattern of co-occurring symptoms. Anxiety, depression, memory impairment, pain, fatigue, and age contributed most to the prediction model, whereas KPS, tumor grade, tumor size, resilience, and perceived social support contributed relatively little in this dataset. Later symptom burden may therefore depend less on conventional clinical descriptors alone than on the combination of distress, cognitive difficulty, and physical discomfort already present at diagnosis (29, 30). Because these variables are routinely available in clinical care, the model may have value for early risk stratification, patient-reported outcome surveillance, and supportive care triage in neurosurgical settings, although its performance will need confirmation in independent cohorts before broader clinical application.

The network findings point in the same direction. Speech difficulty and headache showed the highest strength centrality, and the community structure was overlapping rather than clearly separated. Neurological and arousal-related symptoms clustered together, but depression and headache also showed links across communities. This pattern is not well captured by a symptom-by-symptom approach. Patients presenting with fatigue, sleep disturbance, cognitive complaints, and anxiety are unlikely to benefit from fragmented management in which each symptom is addressed separately. The network is more consistent with integrated supportive care (29–31) and supports multidimensional baseline assessment, coordinated follow-up, and more efficient allocation of symptom-management resources rather than isolated symptom checks. The network analysis and the prediction model therefore address different but related questions: one describes how symptoms are organized at diagnosis, and the other identifies which patients are most likely to remain highly burdened over time.

The prominence of fatigue, sleep disturbance, mood symptoms, and cognitive complaints is broadly consistent with earlier work in oncology and neuro-oncology (32, 33). Cancer-related fatigue has been linked to inflammatory, neuroendocrine, and behavioral mechanisms, and sleep disturbance commonly coexists with psychological distress. In this cohort, affective symptoms were prominent in the prediction model, which is in line with evidence showing that depression and anxiety are common in cancer, including glioma, and may adversely affect adherence, self-management, and health-related quality of life. Cognitive symptoms were likewise important. Memory impairment ranked among the leading predictors, and both memory impairment and attention deficit were closely linked with drowsiness and other neurologically related symptoms. Previous studies have shown (34–36) that cognitive dysfunction in glioma may arise from both disease and treatment and is often present before surgery. In this setting, cognitive complaints are not simply later treatment effects; they are often part of the presenting symptom profile. Emotional distress, fatigue, sleep disturbance, and cognitive difficulty should therefore be recognized early as part of the same clinical picture rather than being addressed in parallel but disconnected ways.

Baseline anxiety and depression were more informative than several disease variables often emphasized in routine documentation. This does not reduce the importance of seizures, headache, or focal deficits, but it does suggest that later symptom burden may be shaped strongly by emotional and cognitive vulnerability in addition to neurological disease burden. Fatigue and pain fit the same pattern. Earlier psychosocial assessment, symptom education, and closer follow-up may therefore be justified for patients who already report substantial distress at diagnosis. Supportive care may need to begin earlier in the treatment course rather than after symptom burden has become established (37, 38). More broadly, a simple baseline framework that relies on routinely collected symptoms may be useful in settings where advanced biomarkers or complex imaging-derived tools are not readily available. This may help explain why a model based on routine baseline symptoms showed good discriminatory performance in the present dataset without relying on specialized biomarkers or advanced imaging features: the symptom burden that unfolds over the following months appears to be foreshadowed, at least in part, by the multidimensional symptom profile already visible at diagnosis. In that sense, routine patient-reported symptom collection may have value not only for individual clinical encounters but also for scalable surveillance and triage pathways.

This prospective study examined baseline symptom structure and its relation to symptom burden at 6 months in the same cohort. The network was most informative in its overall organization and in strength centrality, and these findings were broadly consistent with the prediction model. Symptoms in newly diagnosed glioma were closely interconnected, and anxiety, depression, memory impairment, pain, fatigue, and age emerged as the strongest baseline predictors of high symptom burden at 6 months. Multidimensional assessment at diagnosis may help identify patients at greater risk and support earlier supportive care. Several points should be kept in mind when interpreting these results. This was a single-center study with a modest sample size, and the prediction model still requires external validation. Symptom severity was measured with brief numeric rating scales, which are practical for routine follow-up but less detailed than disease-specific standardized instruments. Treatment variables were excluded from the baseline model so that prediction would rely only on information available at diagnosis, although these factors may influence symptom burden over time. In addition, follow-up was completed using standardized procedures, but formal attrition-bias analyses were not performed in the present study. The model was internally validated only and should be regarded as an exploratory risk-stratification approach rather than a ready-to-implement clinical decision tool. These considerations define the scope within which the findings should be interpreted and generalized.

## Conclusion

Symptom burden in glioma reflects an interconnected symptom pattern rather than a set of isolated complaints. Anxiety, depression, memory impairment, pain, and fatigue contributed substantially to the prediction of high symptom burden at 6 months. Baseline multidimensional assessment may help identify patients who are likely to need closer surveillance and earlier supportive care during the initial treatment course.

## Data Availability

The raw data supporting the conclusions of this article will be made available by the authors, without undue reservation.
